# Apropos: critical analysis of molluscicide application in schistosomiasis control programs in Brazil

**DOI:** 10.1186/s40249-017-0246-x

**Published:** 2017-03-07

**Authors:** Hongjun Li, Wei Wang

**Affiliations:** 1 Weifang Maternity and Child Care Hospital, 407 Qingnian Road, Weicheng District, Weifang City, Shandong Province 261011 China; 2 Key Laboratory of National Health and Family Planning Commission on Parasitic Disease Control and Prevention, 117 Yangxiang, Meiyuan, Wuxi City, Jiangsu Province 214064 China; 3 Jiangsu Provincial Key Laboratory on Parasites and Vector Control Technology, 117 Yangxiang, Meiyuan, Wuxi City, Jiangsu Province 214064 China; 4 Jiangsu Institute of Parasitic Diseases, 117 Yangxiang, Meiyuan, Wuxi City, Jiangsu Province 214064 China; 5 School of Public Health, Fujian Medical University, No. 88 Jiaotong Road, Fuzhou City, Fujian Province 350004 China

**Keywords:** Schistosomiasis, *Oncomelania hupensis*, Molluscicide, 26% suspension concentrate of metaldehyde and niclosamide, 25% suspension concentrate of niclosamide ethanolamine salt, 50% niclosamide ethanolamine salt wettable powder, 4% niclosamide ethanolamine salt dustable powder, 5% niclosamide ethanolamine salt granule, Luowei

## Abstract

Schistosomiasis is a snail-transmitted infectious disease affecting over 200 million people worldwide. Snail control has been recognized as an effective approach to interrupt the transmission of schistosomiasis, since the geographic distribution of this neglected tropical disease is determined by the presence of the intermediate host snails. In a recent Scoping Review published in *Infectious Diseases of Poverty*, Coelho and Caldeira performed a critical review of using molluscicides in the national schistosomiasis control programs in Brazil. They also described some chemical and plant-derived molluscicides used in China. In addition to the molluscicides described by Coelho and Caldeira, a large number of chemicals, plant extracts and microorganisms have been screened and tested for molluscicidal actions against *Oncomelania hupensis*, the intermediate host of *Schistosoma japonicum* in China. Here, we presented the currently commercial molluscicides available in China, including 26% suspension concentrate of metaldehyde and niclosamide (MNSC), 25% suspension concentrate of niclosamide ethanolamine salt (SCNE), 50% niclosamide ethanolamine salt wettable powder (WPN), 4% niclosamide ethanolamine salt dustable powder (NESP), 5% niclosamide ethanolamine salt granule (NESG) and the plant-derived molluscicide “Luowei”. These molluscicides have been proved to be active against *O. hupensis* in both laboratory and endemic fields, playing an important role in the national schistosomiasis control program of China. Currently, China is transferring its successful experiences on schistosomiasis control to African countries. The introduction of Chinese commercial molluscicides to Africa, with adaptation to local conditions, may facilitate the progress towards the elimination of schisosomiasis in Africa.

**Electronic supplementary material:**

The online version of this article (doi:10.1186/s40249-017-0246-x) contains supplementary material, which is available to authorized users.

## Multilingual abstract

Please see Additional file 1 for translations of the abstract into the six official working languages of the United Nations.

## Introduction

Schistosomiasis, caused by the blood fluke parasites of *Schistosoma* genus, is a snail-transmitted infectious disease affecting over 200 million people worldwide [1]. Since the geographic distribution of this neglected tropical disease is determined by the presence of the intermediate host snails of the parasite, snail control is considered to be effective to interrupt the transmission of the disease [2]. A recent quantitative analysis of schistosomiasis control outcomes captured from 83 countries and territories over the past 100 years, showed that the extensive snail control contributed to a reduction of schistosomiasis prevalence by 92%, while the control programs with little or no snail control interventions achieved a 37% reduction, which highlights the critical role of snail control in the schistosomiasis control programs [3].

In a recent Scoping Review published in in the journal *Infectious Diseases of Poverty*, Coelho and Caldeira [4] performed a critical review of using molluscicides in the national schistosomiasis control programs in Brazil. They also described some chemical and plant-derived molluscicides used in China. From the initiation of the national schistosomiasis control program until now, the national schistosomiasis control strategy of China has shifted from transmission control strategy based on snail control between mid-1950s and early 1980s, praziquantel chemotherapy based-morbidity control strategy during the period from mid-1980s through 2003, to the current integrated control strategy since 2004, and snail control has been an important part of the national schistosomiasis control strategy throughout the control programs [5]. Currently, molluscicide treatment remains the predominant approach for snail control in China [6]. In addition to the molluscicides described by Coelho and Caldeira, a large number of chemicals, plant extracts and microorganisms have been screened and evaluated for molluscicidal actions against *Oncomelania hupensis*, the intermediate host of *S. japonicum*. Here, we summarized the currently commercial molluscicides available in China, which have been proved to be active against *O. hupensis* in both laboratory and endemic fields.

### 26% suspension concentrate of metaldehyde and niclosamide (MNSC)

MNSC, a mixture of 25% niclosamide and 1% metaldehyde, is prepared by grinding niclosamide, metaldehyde, glycerol, sodium carboxymethyl cellulose, sodium benzoate and distilled water in a ball miller to yield a suspension formulation. In the laboratory, immersion with MNSC at 0.125 mg/L and higher concentrations for 24 h achieved 100% snail mortality, with a 24 h LC_50_ of 0.058 mg/L and a 48 h LC_50_ of 0.044 mg/L. Spraying with MNSC at 2 g/m^2^ resulted in 94, 100 and 100% snail mortality at 1, 3, and 7 days, respectively [7]. In the marshland and lake regions, the field immersion test showed 97.5–100%, 98.89–100% and 100% snail mortality following treatment with MNSC at 2 g/m^3^ for 24, 48 and 72 h, while the field spraying test revealed 67.81–95.53%, 72.8–89.68%, and 86.67–100% snail mortality following treatment with MNSC at 2 g/m^2^ for 1, 3, and 7 days, respectively [7]. In the mountainous regions, spraying with MNSC at 6 g/m^2^ resulted in 92.88–94.46% snail mortality at 7 days, 90.47–93.79% at 15 days and 88.68–94.27% at 30 day, respectively [8]. Compared with the 50% wettable powder of niclosamide ethanolamine salt (WPN), the first-choice chemical molluscicide used for snail control in China since 1990s on the recommendation of WHO [9], MNSC reduces the toxicity of niclosamide to the environment and fish [7]; however, the exact effect of MNSC on the environment and the mechanisms underlying the molluscicidal efficacy of the drug against *O. hupensis* snails remain to be investigated.

In addition, MNSC was reported to be active against *Lymnaea* species [10], and 25% suspension concentrate of niclosamide (SCN) has shown toxicity to both adult and juvenile *Biomphalaria glabrata* [11]. These exciting findings encourage the investigations of the molluscicidal efficacy of 26% MNSC against *Biomphalaria* spp. and *Bulinus* spp..

### 25% suspension concentrate of niclosamide ethanolamine salt (SCNE)

SCNE is a novel suspension formulation of niclosamide which contains 25% niclosamide ethanolamine salt. In the laboratory, immersion with SCNE at 0.125 mg/L and higher doses for 24 h and a longer period of time resulted in 100% snail mortality, with 0.058, 0.044 and 0.039 mg/L for 24, 48, and 72 h LC_50_ values, respectively, and spraying with SCNE at 1 to 8 g/m^2^ achieved 90–100% snail mortality at 1 day, 97–100% at 3 days, and 99–100% at 7 days, respectively, depending on drug doses [12]. In a marshland and lake region, the field immersion test showed 100% snail mortality following the treatment with SCNE at 2 g/m^3^ for 24, 48, or 72 h, and the field spraying test revealed 74.51–88.81% snail mortality at 1 day, 80.34–98.26% at 3 days and 87.38–100% at 7 days depending on the drug doses given (1 to 8 g/m^2^) [12]. This formulation provides a novel choice of chemical molluscicides for the field application in the endemic foci of China; however, further studies are required to investigate the environmental toxicity and the mechanisms of action of SCNE.

### WPN

With the initiation of the World Bank Loan Project for Schistosomiasis Control in China since 1992, WPN was introduced and currently it is the most widely used chemical molluscicide in China [13]. The sensitivity of *O. hupensis* to WPN varies in the schistosomiasis-endemic regions of China [14], and a recent meta-analysis showed 77% (95% *CI*: 68 to 86%), 83% (95% *CI*: 77 to 89%) and 88% (95% *CI*: 82 to 92%) snail mortality after spraying WPN at a dose of 2 g/m^2^ for 3, 7 and 15 days in the marshland regions [15]. Excitingly, no evidence of resistance to WPN has been detected in *O. hupensis* after more than 2 decades of repeated, extensive application in the main endemic foci of China [16].

Recently, de Novo transcriptome analysis revealed 254 differentially expressed unigenes in WPN-treated *O. hupensis* snails, which were associated with cell structure defects and inhibition of neurohumoral transmission and energy metabolism that may cause snail death [17]. In addition, WPN treatment resulted in damages to cell structures and organelles of *O. hupensis* snails, suggesting the suppression of the movement ability and effects on liver and energy metabolism; in parallel, activities of carbohydrate metabolism-associated enzymes were inhibited, and activities of stress response-associated enzymes were increased followed by a reduction to lower levels than those of the H_2_O-treated group [18]. This shift of carbohydrate metabolism patterns led to insufficient energy supply and lactic acid accumulation [18]. Variations of nitric oxide synthase (NOS), alanine aminotransferase (ALT), and superoxide dismutase (SOD) during the WPN treatment suggested a stress response of *O. hupensis* snail to the molluscicide at early stages and later fatal damage in the liver and the nervous system [18]. However, the environmental issues related to the use of WPN have not been clearly demonstrated to date.

### 4% niclosamide ethanolamine salt dustable powder (NESP)

Although WPN is highly active against *O. hupensis*, the use of this niclosamide formulation requires water, which limits its application in the endemic regions lacking water [19]. To overcome this problem, a novel formulation of niclosamide, NESP, was developed in 2003, which contains 4% niclosamide ethanolamine salt [20]. Laboratory test showed a 0.197 g/m^2^ 24 h LC_50_ for NESP against *O. hupensis* [21], and the meta-analysis showed 81% (95% *CI*: 65 to 93%), 90% (95% *CI*: 83 to 95%) and 94% (95% *CI*: 91 to 97%) snail mortality after treatment with NESP at a 2 g/m^2^ used dose of niclosamide for 3, 7 and 15 days in the marshland regions [15]. NESP has been considered an effective supplement of currently available chemical molluscicides in China [22]; however, the powder may drift, which may result in potential environmental pollution.

### 5% niclosamide ethanolamine salt granule (NESG)

The use of niclosamide suspension formulations requires water, and the powder formulation suffers from the drug drift and the resultant environmental pollution, as well as potential damages to workers spraying the powder. NESG was therefore developed in order to overcome these problems [23]. Laboratory test showed that spraying with NESG at 0.5, 1 and 2 g/m^2^ resulted in 85–98% snail mortality at 1 day, 93–99% at 3 days and 96–100% at 7 days, respectively, depending on drug doses [23]. In the marshland region, 7 days treatment with 2 g/m^2^ NESG achieved 85.42% snail mortality and a 93.1% reduction in the density of living snails [24], and in the mountainous region, spraying with NESG at 2 g/m^2^ resulted in 72.41% snail mortality at 7 days, 74.61% at 15 days and 73.24% at 30 day, and reduced the density of living snails by 72.26% at 7 days, 74.09% at 15 days and 75.91% at 30 day, respectively [25]. NESG is a breakthrough niclosamide formulation of chemical molluscicides in China, and this agent greatly expands the application range of niclosamide formulations. Further studies to investigate the environmental toxicity of this formulation seem justified.

### “Luowei”, a plant-derived molluscicide

“Luowei” is a pentacyclic triterpenoid extracted from the seed of the plant *Camellia oleifera*. Laboratory immersion test showed 93.33, 96.67 and 100% snail mortality at 24, 48 and 72 h after treatment with “Luowei” at a dose of 2.5 g/L, and 96.67, 100 and 100% snail mortality at 24, 48, and 72 h after treatment with “Luowei” at 5 g/L, and laboratory spraying test revealed 70, 76.67 and 83.33% snail mortality at 1, 3 and 7 days following treatment with “Luowei” at 2.5 g/m^2^, and 90, 96.67, and 100% snail mortality at 1, 3, and 7 days following treatment with “Luowei” at 5 g/m^2^ [26]. In addition, the field test showed that spraying with “Luowei” at 5 g/m^2^ achieved 81.57% snail mortality at 1 day, 84.98% at 3 days, 84.68% at 7 days, and 86.53% at 15 days in the marshland and lake regions [27]; 81.44% snail mortality at 1 day, 86.95% at 3 days, 90.3% at 7 days, and 90.26% at 15 din the plain regions [28]; 56.47% snail mortality at 1 day, 57.32% at 3 days, 90.58% at 7 days and 93.41% at 15 days in the hilly regions [29]; and 69.55% snail mortality at 1 day, 78.09% at 3 days, 88.39% at 7 days and 87.39% at 15 days in the mountainous regions, respectively [30]. Toxicity testing showed a 0.15 mg/L (95% *CI*: 0.14 to 0.17 mg/L) LC_50_ for “Luowei” in the Zebra fish, > 60 mg/kg LC_50_ in *Coturnix coturnix*, a 6.28 mg/L (95% *CI*: 3.53 to 11.2 mg/L) 96 h LC_50_ in the freshwater shrimps [31], and an acute inhalation LC_50_ of >5000 mg/m^3^ in rats [32]. However, the mechanisms of action and environmental assessment of this plant-derived molluscicide have not been reported until now.

In addition, “Luowei” was found to be active against *Pomacea canaliculata* [33], and the molluscicidal efficacy of “Luowei” against *Biomphalaria* and *Bulinus* is under evaluation.

## Conclusions

During the past two decades, a large number of novel molluscicides have been screened and developed in China, and many of them have been made commercial. In addition to chemical and plant-derived agents, the molluscicidal action of microorganisms against *O. hupensis* has been recently paid more and more attention [34–36].

Currently, China is transferring its successful experiences on schistosomiasis control to African countries [37, 38]. The introduction of Chinese commercial molluscicides, with adaptation to African local conditions, may facilitate the progress towards the elimination of schisosomiasis in Africa [39].

## Additional file

Additional file 1: Multilingual abstract in the six official working languages of the United Nations. (PDF 688 kb)

## Abbreviations

MNSC: 26% suspension concentrate of metaldehyde and niclosamide
NESG: 5% niclosamide ethanolamine salt granule
NESP: 4% niclosamide ethanolamine salt dustable powder
SCN: 25% suspension concentrate of niclosamide
SCNE: 25% suspension concentrate of niclosamide ethanolamine salt
WPN: 50% niclosamide ethanolamine salt wettable powder
