# Supplementary material for: Apropos: critical analysis of molluscicide application in schistosomiasis control programs in Brazil
Source: Infect Dis Poverty. 2017 Mar 7;6:54. doi: 10.1186/s40249-017-0246-x (PMC5341408; doi:10.1186/s40249-017-0246-x)
Supplement: Additional file 1: — Multilingual abstract in the six official working languages of the United Nations. (PDF 688 kb) [file 40249_2017_246_MOESM1_ESM.pdf]

Translation of the abstract into the six official working languages of the United Nations

المقترح: دراسة تحليلة لإستخدام مبيدات الرخويات في برامج مكافحة البلهارسيا في البرازيل.

هونجن لي و وي وانق

الملخص
البلهارسيا هى مرض معدى ينتقل بواسطة القواقع ويقدر عدد المصابين به في العالم بأكثر من 200 مليون شخص. و تعتبر مكافحة القواقع طريقة فعاله للحد من إنتقال البلهارسيا حيث أن التوزيع الجغرافي لهذا المرض الإستوائي المهمل يحدد بظهور القواقع التي تعمل كعامل وسيط. وفي دراسة نشرت حديثا في دورية ***الامراض المعدية الناتجةمن الفقر*** أجرى كويلهو وكالديرا مراجعه نقدية لإستخدام مبيدات الرخويات في برنامج البرازيل القومي لمكافحة البلهارسيا. ووصفوا أيضا بعض المبيدات الكيميائية والمستخلصة من النباتات المستخدمة في الصين.بالإضافة إلى المبيدات التي وصفت من قبل كويلهو وكالديرا, تم فحص عدد كبير من المواد الكيميائية و المستخلصات النباتية و الكائينات الدقيقة وأختبرت لإبادة القواقع التي هي العائل الوسيط لطفيل البلهارسيا في الصين. ونقدم هنا مبيدات الرخويات التجارية المتوفرة الأن في الصين , تتضمن 26% من تركيز المعلق ميتالدهيد ونيكلوساميد (MNSC), 25% من تركيز المعلق ملح النيكلوساميد إثانولاماين (NESP), و 50% من مسحوق ملح النيكلوساميد ل إثانولاماين القابلة للبلل (WPN), و 4% من مسحوق ملح النيكوساميد إثانولاماين الترابي (NESP) ,و 5% من حبيبات ملح النيكوساميد إثانولاماين والمبيد المستخلص من النبات" ليووي". وقد أثبتت هذه المبيدات فعاليتها ضد قواقع البلهارسيا في المعامل و الحقول المستوطنة لاعبة دورا هاما في البرنامج القومي لمكافحة البلهارسيا في الصين .وتنقل الصين حاليا تجاربها الناجحة في مكافحة البلهارسيا للدول ألافريقية.إن إدخال مبيدات الرخويات التجارية الصينية لأفريقيا, مع مواءمتها للاحوال المحلية , قد تسهل التقدم نحو القضاء على البلهارسيا في أفريقيا.

Translated from English version into Arabic by Shaza Swareldhab, through

TRANSLATORS
WITHOUT BORDERS

读“灭螺药物在巴西血吸虫病防治规划中应用的思辨分析”有感

李洪军，汪伟

摘要
血吸虫病是一种呈世界性分布的螺传传染病，全球感染者超过 2 亿。流行病学研究表明，血吸虫病的地理分布与中间宿主螺的分布区高度一致。因此，消灭钉螺是阻断血吸虫病传播的有效措施之一。近期发表在《贫困所致传染病（英文）》上的一篇勘域综述中，巴西学者 Coelho PMZ 和 Caldeira RL 对灭螺药物在巴西血吸虫病防治规划中的应用进行了思辨分析。此外，他们还介绍了一些在中国应用的化学和植物灭螺药。事实上，除了巴西学者在该综述中介绍的灭螺药外，近年来中国学者筛选了大量化学、植物和微生物灭螺药，并对其杀灭日本血吸虫中间宿主湖北钉螺的效果进行了评价。本文主要对目前在中国血吸虫病流行区广泛应用的商品化灭螺药物作一简要概述，包括 26%四聚•杀螺胺悬浮剂（MNSC）、25%氯硝柳胺乙醇胺盐悬浮剂（SCNE）、50%氯硝柳胺乙醇胺盐可湿性粉剂（WPN）、4%氯硝柳胺乙醇胺盐粉剂（NESP）、5%杀螺胺乙醇胺盐颗粒剂（NESG）和植物灭螺药“螺威”。实验室和现场研究证实，这些商品化灭螺药物具有较好的灭螺效果，在中国血吸虫病防治规划中发挥了重要作用。目前，中国正在将血吸虫病防控方面取得的成功经验输出到非洲国家，以帮助其消除血吸虫病的危害。如能将这些商品化灭螺药物引入到非洲，并因地制宜加以应用，可能会促进非洲血吸虫病消除进程。

Translated from English version into Chinese by Wei Wang

A propos: Analyse critique de l’utilisation de molluscicides dans les programmes de contrôle de la schistosomiase au Brésil

Hongjun Li and Wei Wang

Résumé: La schistosomiase est une maladie infectieuse transmise par un mollusque aquatique ; plus de deux cents millions d’individus sont affectés par la maladie à travers le monde. Le contrôle du mollusque a été reconnu comme une méthode efficace pour interrompre la transmission de la schistosomiase, depuis que la propagation de cette maladie tropicale oubliée a été identifiée par la présence d’escargots considérés comme hôtes intermédiaires. Dans une récente étude préliminaire publiée dans *Maladies infectieuses de la pauvreté*, Coelho et Caldeira ont effectué une analyse critique concernant l’utilisation des molluscicides dans les programmes de contrôle nationaux de la schistosomiase au Brésil. Ils ont aussi évoqué l’utilisation de

molluscicides chimiques et d'origine végétale en Chine. Outre les molluscicides désignés par Coelho et Caldeira, un grand nombre de produits chimiques, d'extraits de plantes et de micro-organismes ont été examinés et testés pour leurs principes atifs molluscicide pour combattre *Oncomelania hupensis*, l'hôte intermédiaire du *Schistosoma japonicum* en Chine. Voici les molluscicides actuellement commercialisés et disponibles en Chine, incluant 26% de concentrés de métaldéhyde et de niclosamide en suspension (MNSC), 25% de concentré de sel de niclosamide éthanolamine (SCNE), 50% de sel de niclosamide éthanolamine sous forme de poudre mouillable (WPN), 4% de sel de niclosamide éthanolamine sous forme de préparation en poudre (NESP), 5¨% de sel de niclosamide éthanolamine sous forme de granulés (NESG) et un molluscicide d'origine végétale «Luowei». Ces molluscicides se sont avérés efficaces pour combattre *O.hupensis* tant dans les laboratoires que dans les secteurs endémiques, jouant ainsi un rôle important dans le programme de contrôle national de la schistosomiase de Chine. Actuellement la Chine fait part de ses expériences concluantes sur le contrôle de la schistosomiase à tous les pays d'Afrique. Introduire le commerce des molluscicides chinois en Afrique, (en les adaptant aux conditions locales) peut faire avancer l'élimination de la schistosomiase dans ce continent.

Translated from English version into French by veromarie, through

TRANSLATORS WITHOUT BORDERS

Касательно: Критического анализа применения моллюскицидов в рамках программы по контролю над бильгарциозом в Бразилии

Хонгджун Ли и Вэй Ванг

Резюме

Бильгарциоз является инфекционным заболеванием, передаваемым брюхоногими моллюсками, поражающим более 200 миллион человек по всему миру. Так как географически распространение данного, обделенного вниманием, тропического заболевания обусловлено наличием моллюсков, выступающих промежуточными хозяевами, контроль над моллюсками признан эффективным методом по предотвращению передачи бильгарциоза. В недавнем Обзоре опубликованном в «Инфекционных болезнях бедности» Коэльо и Калдейра критически рассмотрели применение моллюскицидов в рамках национальной программы по контролю над бильгарциозом в Бразилии. Они также дали описание некоторых химических моллюскицидов и моллюскицидов растительного происхождения, применяемых в Китае. Кроме моллюскицидов, описанных Коэльо и Калдейра, большое количество химикатов, растительных экстрактов и микроорганизмов было исследовано на моллюскицидную активность в отношении *Oncomelania hupensis*, промежуточного хозяина *Schistosoma japonicum* в Китае. Здесь мы представляем коммерческие моллюскициды, доступные в настоящее время в Китае, включая 26%-ый концентрат суспензии метальдегида и никлозамида (MNSC), 25%-ый концентрат суспензии соли никлозамид этаноламина (SCNE), 50%-ый смачиваемый порошок соли никлозамид этаноламина (WPN), 4%-ый распыляемый порошок соли никлозамид этаноламина (NESP), 5%-ую гранулированную соль никлозамид этаноламина (NESG) и моллюскицид растительного происхождения "Luowei". Данные моллюскициды признаны эффективными против *O. hupensis* как в лабораторной, так и в эндемичной среде, играя важнейшую роль в национальных программах по контролю над бильгарциозом в Китае. В настоящее время Китай передает свой успешный опыт по контролю над бильгарциозом Африканским странам. Представление китайских коммерческих моллюскицидов в Африке, адаптированных к местным условиям, позволило бы предотвратить развитие бильгарциоза в Африке.

Translated from English version into Russian by Ms Zhdanova, through

TRANSLATORS WITHOUT BORDERS

Asunto: Análisis crítico de la aplicación de molusquicidas en los programas de control de la esquistosomiasis en Brasil

Hongjun Li y Wei Wang

Resumen

La esquistosomiasis es una enfermedad infecciosa, transmitida por caracoles, que afecta a más de 200 millones de personas en todo el mundo. Se ha reconocido que el control de caracoles es un método efectivo para interrumpir la transmisión de la esquistosomiasis, ya que la distribución geográfica de esta enfermedad tropical desatendida viene determinada por la presencia de los caracoles anfitriones intermediarios. En una reciente revisión exploratoria publicada en *Infectious Diseases of Poverty*, Coelho y Caldeira hacen un análisis crítico del uso de molusquicidas en los programas nacionales de control de la esquistosomiasis en Brasil, y describen algunos molusquicidas obtenidos a base de productos químicos y plantas utilizados en China. Además de los molusquicidas descritos por Coelho y Caldeira, se ha revisado y analizado un amplio número de productos químicos, extractos de plantas y microorganismos contra el *Oncomelania hupensis*, el anfitrión intermediario del *Schistosoma japonicum* en China. Aquí presentamos los molusquicidas comercializados actualmente en China, como un 26 % de suspensión concentrada de metaldehído y niclosamida (SCMN), un 25 % de suspensión concentrada de sal de etanolamina niclosamida (SCEN), un 50 % de polvo humectable de sal de etanolamina niclosamida (PHEN), un 4 % de polvo seco de sal etanolamina niclosamida (PSEN), un 5 % de gránulos de sal etanolamina niclosamida (GSEN) y molusquicida obtenido a base de plantas "Luowei". Estos molusquicidas han demostrado ser activos contra el *O. hupensis* tanto en el laboratorio como en los campos endémicos, de modo que ha jugado un importante papel en el programa nacional de control de la esquistosomiasis de China. En la actualidad, China está transmitiendo sus experiencias sobre el control de la esquistosomiasis a los países africanos. La introducción de molusquicidas comerciales chinos en África, con la adaptación a las condiciones locales, puede facilitar el progreso hacia la eliminación de la esquistosomiasis en África.

Translated from English version into Spanish by Mercedes Pacheco, through

TRANSLATORS
WITHOUT BORDERS
